# Insight into the genome data of commercially important giant kelp *Macrocystis pyrifera*

**DOI:** 10.1016/j.dib.2022.108068

**Published:** 2022-03-18

**Authors:** Sujay Paul, Erika Salavarría, Katherine García, Alonso Reyes-Calderón, Patricia Gil-Kodaka, Ilanit Samolski, Aashish Srivastava, Anindya Bandyopadhyay, Gretty K. Villena

**Affiliations:** a Tecnologico de Monterrey, School of Engineering and Sciences, Campus Queretaro, Av. Epigmenio Gonzalez, No. 500 Fracc. San Pablo, Queretaro CP 76130, Mexico; b Facultad de Ciencias del Mar, Grupo de investigación ‘Bioeconomia Costera’, Universidad Estatal Península de Santa Elena, Km 1 Sta. Elena – La Libertad. Ecuador; c Laboratorio de Micología y Biotecnología LMB, Universidad Nacional Agraria La Molina, Av. La Molina s/n, 12, Lima, Peru; d Facultad de Pesquería, Universidad Nacional Agraria La Molina, Lima, Peru; e Department of Clinical Science, University of Bergen, Bergen 5021, Norway; f International Rice Research Institute, Manila 4031, Philippines; g Reliance Industries Ltd., Navi Mumbai 400701, India

**Keywords:** Brown alga, *Macrocystis pyrifera*, Valuable bioproducts, Whole genome sequencing, Illumina, Nanopore

## Abstract

Kelps or brown algae are a wide group of marine macroalgae that play an important role in aquatic ecosystems and generally have high commercial value. To facilitate brown algal studies, we report the complete genome sequence of the largest kelp *Macrocystis pyrifera*. The whole genome is ∼428 Mb in size, comprises 44,307 scaffolds with an average GC content of 47%, and is predicted to contain a total of 24,778 genes. 18S sequence-based phylogenetic analysis revealed that littoral brown seaweed *Scytosiphon lomentaria* is the closest species of *M. pyrifera*. Numerous genes identified in this dataset are involved in genetic information processing, signaling, and cellular processes, carbohydrate metabolism, and terpenoids biosynthesis.

## Specifications Table

**Table:** 

Subject	Genomics
Specific subject area	Algal Genomics
Type of data	Tables, Figures, Charts
How the data were acquired	Illumina HiSeq 4000 (paired-end) and Nanopore GridIon-X5
Data format	Raw, filtered, analyzed
Description of data collection	Genomic DNA was extracted and purified from apical frond tissue samples of Macrocystis pyrifera using Gene Jet Plant genomic DNA purification Kit (Thermo Scientific, USA) and sequenced both on Illumina Hiseq 4000 (paired-end) and Nanopore- GridION platforms. The short reads (Illumina) and long reads (Nanopore) data from both the sequencing platforms were demultiplexed using bcl2fastq (Illumina) and guppy (Oxford Nanopore Technologies). *De novo* hybrid assembling was done with MaSuRCA software.
Data source location	Punta San Juanito, Ica, Peru (Latitude 15°15′11.3′′S, Longitude 0.75°13′ 32.4′′W)
Data accessibility Repository name	The nucleotide sequences of raw reads and assembled draft genome are available at NCBI's Sequence Read Archive as BioProject PRJNA605694 (https://www.ncbi.nlm.nih.gov/bioproject/PRJNA605694)

## Value of the Data

• This is a high-quality draft genome sequence report of the commercially valuable largest brown algae, which will aid macroalgal genome research.
• The draft genome data facilitates identifying several genes involved in the biosynthesis of industrially important complex algal cell wall polysaccharides, which will be useful for polymer biologists or researchers from biochemical industries to develop innovative products.
• The draft genome data facilitates identifying a number of terpenoid biosynthetic genes, which will help understand the terpenoid metabolism in macroalga and accelerate marine bioprospecting.
• The draft genome data will boost macroalgal functional genomics studies.

## 1 Data Description

**Fig. 1:**
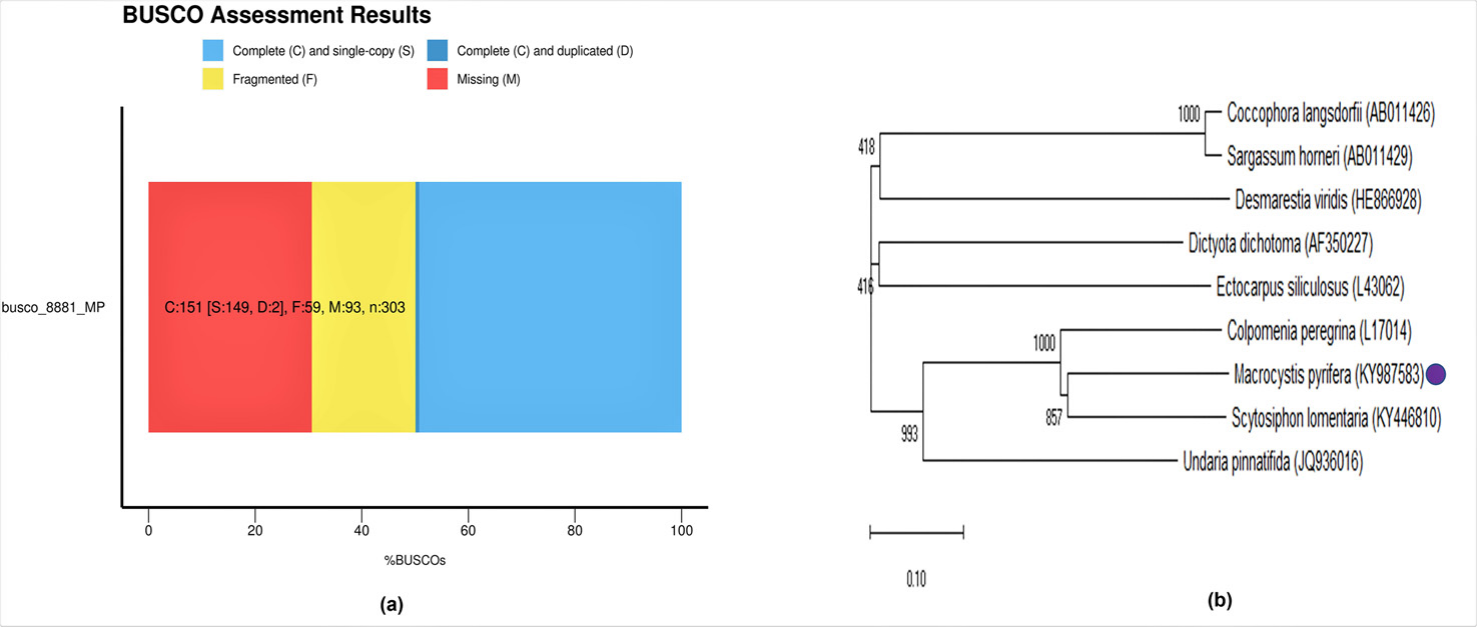
(a) BUSCO evaluation of completeness of *M. pyrifera* genome. (b) 18S sequence-based phylogenetic analysis revealed littoral brown seaweed *Scytosiphon lomentaria* as the closest species of *M. pyrifera*. The phylogenetic tree was constructed using MEGA-X v10.0.5 tools through the maximum likelihood method. Bootstrap analysis (1000 replicates) was performed to validate the nodes.

Kelps or brown algae (Chromista, Phaeophyceae) are multicellular photosynthetic organisms that play crucial roles in the marine ecosystem. Giant kelp *Macrocystis pyrifera* is the largest and most commonly distributed kelp species on the planet, making it one of the richest ecosystems on earth [1] that is often cultured commercially for marine bioprospecting (developing commercially valuable products exploiting marine organisms) [2]. The economic importance of *M. pyrifera* is primarily due to its use in the industrial production of the high-value complex polysaccharides alginic acids /alginates and sulfated fucans /fucoidans, although other valuable carbohydrates such as mannitol and laminarin as well as biochemical compounds are also extracted from this kelp species [3,4]. A total of 44.64 million paired-end reads were generated from Illumina and 4 million reads from Nanopore-GridION, respectively. The scaffolded *M. pyrifera* genome was of size ∼428 Mb with an N50 size of ∼42.8 Kb and contained 44,307 scaffolds (Table 1). The GC content was calculated as ∼47%. A total of 24,778 genes and 20,026 annotated proteins were predicted in the analysis. Genes with Pfam domains and signal peptides were found to be 9,705 and 521 in number, respectively. The average lengths (bp) of the gene, coding sequence, and intron of the *M. pyrifera* genome were found to be ∼5,949, ∼978, and ∼1,514, respectively (Table 1), and the BUSCO evaluation of completeness of the genome was ∼50% complete (Table 1, Fig. 1a). The phylogenetic tree displayed that littoral brown seaweed *Scytosiphon lomentaria* is phylogenetically the closest species of *M. pyrifera* (Fig. 1b).

**Table 1:** General features of *M. pyrifera* the genome.

Genome size (bp)	427,916,191
DNA coding (bp)	3,868,356
GC Contents (%)	47.36
Total assembled length (bp)	427,916,191
Number of DNA scaffolds	44,307
N50 scaffold size	42,778 (42.8 Kb)
Number of total genes	24,778
Number of total annotated proteins	20,026
Genes with Pfam domains	9,705
Genes with signal peptides	521
Repeated sequences (values and %)	160,114,011 bp (37.42%)
Average gene length (bp)	5,949
Average coding sequence length (bp)	978
Average intron length (bp)	1,514
Average protein Length	326
Busco Completeness (%)	49.9

**Fig. 2:**
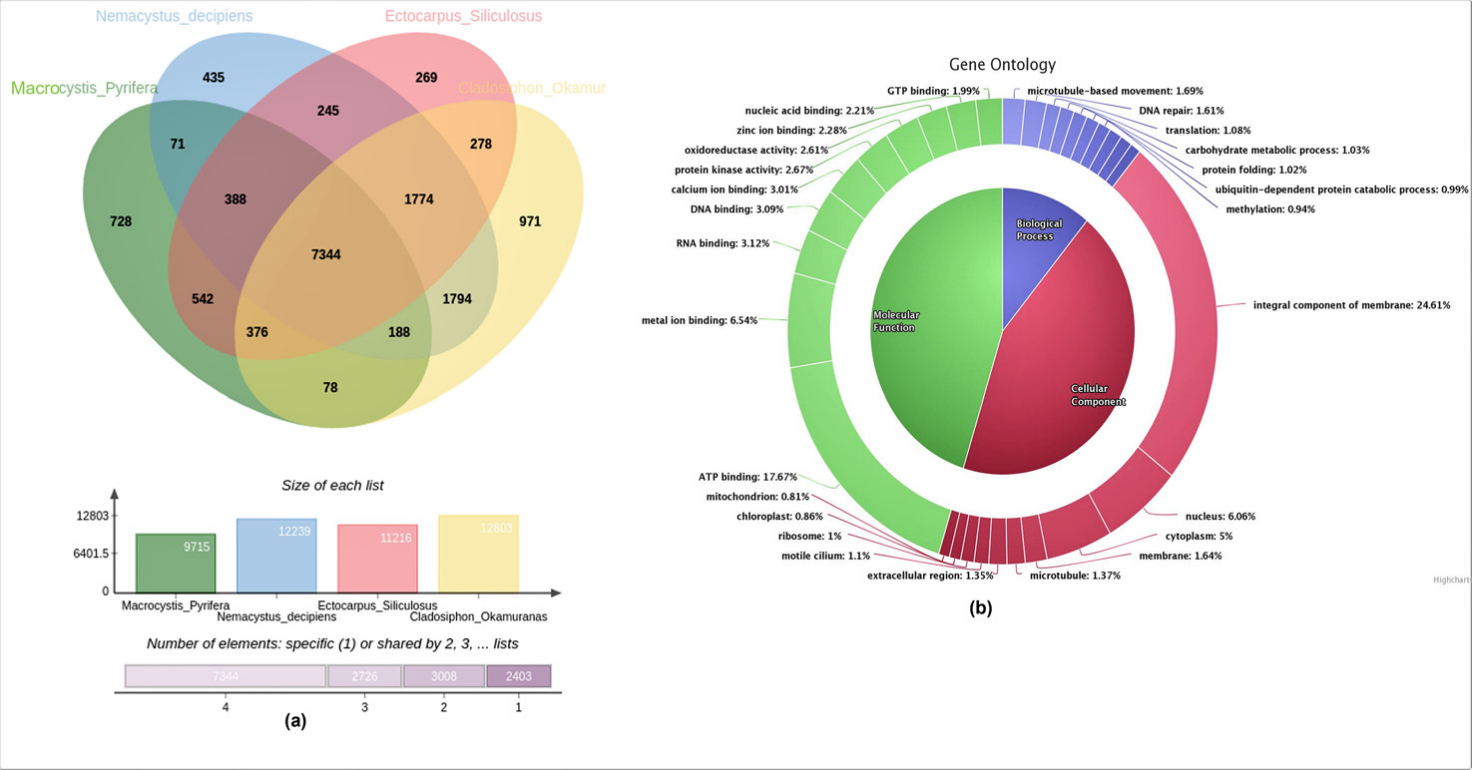
(a) Protein level comparative analysis of *M. pyrifera* against multiple related species such as *Ectocarpus siliculosus, Nemacystus decipiens, and Cladosiphon okamuranus*, using Orthovenn tool. (b) GO enrichment analysis of annotated proteins from *M. pyrifera*.

Protein level comparative analysis using the Orthovenn tool provides information about a list of paralogous and orthologous proteins shared between multiple related species (Fig. 2a). GO analysis of annotated proteins from *M. pyrifera* showed that in the ‘Molecular function’ (MF) category, the highest represented GO term was ATP binding (17.67%) followed by metal ion binding (6.54%) and RNA binding (3.12%), while in the ‘Biological Process’ (BP) category, microtubule-based movement (1.69%) was the highest term followed by DNA repair (1.61%) and translation (1.08%). In the ‘Cellular Component’ category, most of the proteins were found to be an integral component of the membrane (24.61%), followed by the nucleus (6.06%) and cytoplasm (5.0%) (Fig. 2b). Moreover, several important terpenoid biosynthetic genes such as beta-ring hydroxylase, farnesyl-diphosphate farnesyltransferase, phytoene desaturase, transketolase, farnesyl diphosphate synthase, hydroxymethylglutaryl-CoA synthase, squalene monooxygenase, zeta-carotene desaturase, and geranyl diphosphate synthase were identified which could aid in understanding the terpenoid metabolism in macroalga and facilitates marine bioprospecting.

Seaweeds have proved to be of great interest to the pharmaceutical and food industries due to their complex cell wall polysaccharides such as alginates, fucoidans, laminarin, and mannitol. We have identified several complex polysaccharide biosynthetic genes such as Alpha-(1,6)-fucosyltransferase, GDP-mannose 4,6-dehydratase, GDP-fucose pyrophosphorylase, Aryl sulfotransferase, Mannose-6-phosphate isomerase, Phosphomannomutase, GDP-mannose dehydrogenase, Mannuronan C-5-epimerase, and Polyketide Synthase III for alginates and fucoidans biosynthesis; UTP-glucose-1-phosphate uridylyltransferase, UDP-glucose pyrophosphorylase/phosphoglucomutase, 1,3 beta-glucan synthase, Cellulose synthase, Glucose-6-phosphate isomerase, Phosphoglycerate mutase, Trehalose 6-phosphate synthase, and Trehalose 6-phosphate phosphatase for laminarin biosynthesis; and finally Mannitol 1-phosphate dehydrogenase and Mannitol-1-phosphatase for the biosynthesis of mannitol. DNA transposons such as Helitrons and Retrotransposons included LTR (long terminal repeat), Gypsy, and Copia were also identified in this work.

## 2 Experimental Design, Materials and Methods

### 2.1 Sample preparation and sequencing

Apical frond tissue samples of adult *Macrocystis pyrifera* were collected from the intertidal zone (water surface/0 m depth) at Punta San Juanito, Ica, Peru (Latitude 15°15′11.3′′S, Longitude 0.75°13′ 32.4′′W) during low tide, washed subsequently with 100% ethanol and frozen immediately in liquid nitrogen until further analysis. Genomic DNA was extracted and purified from frozen frond tissues using Gene Jet Plant genomic DNA purification Kit (Thermo Scientific, USA) following the manufacturer's protocols. A high-quality DNA sample was used for sequencing, and the whole genome sequencing approach was taken using both Illumina Hiseq 4000 (Illumina) and GridION-X5 (Oxford Nanopore Sequencing Technology) platforms at Genotypic Technology Pvt Ltd (Bengaluru, India).

### 2.2 De novo genome assembly and assessment

The short reads (Illumina) and long reads (Nanopore) data were demultiplexed applying bcl2fastq-v2.17.1.14 and Guppy-v2.3, respectively [5]. *De novo* hybrid assembly was performed by MaSuRCA-v3.3.4 [6] utilizing Illumina and nanopore reads. Scaffolding was done by pyScaf-v0.12a (https://bioinformaticsonline.com/bookmarks/view/30236/pyscaf) to improve the quality of the assembled genome. Further soft masking of the repeat regions found in the draft scaffolded genome was carried out using RepeatMasker-v4.0.6 (https://www.repeatmasker.org/) and RepeatModeler-v2.0 [7]. The improved assembled genome was then employed for predicting the genes and protein sequences applying the BRAKER gene prediction tool [8]. Along with scaffolded genome, reference protein data from *Ectocarpus siliculosus* and transcriptome data (NCBI SRAs: SRR3544557 and SRR3615022) from *M. integrifolia* as well as *M. pyrifera* [9] was used for predicting genes/proteins efficiently. The completeness of scaffolded genome predicted genes and transcriptomes was assessed further using BUSCO-v3.0.2 [10]. The phylogenetic position of *M. pyrifera* was determined using the MEGA-X-v10.0.5 [11] tool through the maximum likelihood method (Jukes-Cantor model); since the whole genome sequences of brown algal species are limited in the NCBI database 18S rDNA sequences of *M. pyrifera* and other kelps (*Coccophora langsdorfii, Sargassum horneri, Dictyota dichotoma, Desmarestia viridis, Ectocarpus siliculosus, Colpomenia peregrina, Undaria pinnatifida*, and *Scytosiphon lomentaria*) were selected to construct the phylogenetic tree. Protein level comparative analysis was carried out among predicted proteins from the sample and reference protein sequences from multiple related species such as *Ectocarpus siliculosus, Nemacystus decipiens, and Cladosiphon okamuranus*. Finally, the Gene Ontology (GO) annotation of the predicted proteins was accomplished via the DIAMOND-v0.8.29 [12] BlastP program against the Uniprot brown algae family protein database.

## Ethics Statement

Not applicable.

## CRediT Author Statement

**Sujay Paul:** Conceptualization, Methodology, Investigation, Writing – original draft; **Erika Salavarría:** Investigation, Formal analysis, Writing – review & editing; **Katherine García:** Formal analysis; **Alonso Reyes-Calderón:** Formal analysis. **Patricia Gil-Kodaka:** Validation, Formal analysis; **Ilanit Samolski:** Writing – review & editing; **Aashish Srivastava:** Formal analysis; **Anindya Bandyopadhyay** Writing – review & editing; **Gretty K. Villena:** Conceptualization, Review & Supervision.

## Declaration of Competing Interest

The authors declare that there are no competing interests.

## Data Availability

BioProject PRJNA605694 (Original data) (NCBI, SRA).
